# Association and interaction between dietary patterns and gene polymorphisms in Liangshan residents with hyperuricemia

**DOI:** 10.1038/s41598-021-04568-y

**Published:** 2022-01-25

**Authors:** Tingting Li, Shuangjing Li, Tian Tian, Zhichao Nie, Wangdong Xu, Longjian Liu, Hong Jia

**Affiliations:** 1 School of Public Health, Southwest Medical University, Luzhou, Sichuan China; 2 Western Theater Command Air Force Hospital, Chengdu, Sichuan China; 3 Nursing Department, Yaan Vocational and Technical College, Yaan, Sichuan China; 4 Department of Epidemiology and Biostatistics, Dornsife School of Public Health, Drexel University, Philadelphia, PA USA; 5 Center for Evidence-based Medicine, Southwest Medical University, Luzhou, China

**Keywords:** Genetics, Diseases, Nutrition

## Abstract

Hyperuricemia (HUA) is associated with dietary and genetic factors. However, studies on dietary patterns and their interaction effect with genes on the risk of HUA are limited. We aimed to explore the association between dietary patterns and HUA, and dietary patterns—gene interactions on the risk of HUA. A population-based cross-sectional study was conducted in adults aged 18 and older in Liangshan Yi Autonomous Prefecture of China. Dietary consumption was collected using a standard Food Frequency Questionnaire. Vein blood samples were collected after overnight fasting, and DNA was extracted from peripheral blood leukocytes. Dietary patterns were derived using principal component and factor analysis. Of the 2646 participants, the prevalence of HUA was 26.8%. Three dietary patterns were classified. Of them, a dietary pattern with higher meat consumption (defined as meat-based) had the strongest association with HUA than a dietary pattern with plant-based or local special diet-based. A higher frequency of T allele at *ABCG2* rs2231142 and *SLC2A9* rs11722228 loci was observed in participants with HUA than those without HUA. An additive interaction of meat-based dietary pattern with rs2231142 locus was significantly associated with an increased risk of HUA. The relative excess risks of interaction, attributable proportion of interaction, and synergy index (S) were 0.482 (95% CI: 0.012–0.976), 0.203 (95% CI: 0.033–0.374), and 1.544 (95% CI: 1.012–2.355), respectively. In conclusion, a dietary pattern with meat-based was significantly associated with an increased risk of HUA. There was an additive interaction between a meat-based dietary pattern and the *ABCG2* rs2231142 locus. Individuals with rs2231142 T allele were at higher risk of HUA than those with rs2231142 GG allele.

## Introduction

Hyperuricemia (HUA) is a purine metabolic disorder. The most well-known disease induced by HUA is gout, but many studies have reported that HUA also plays an important role in cardiac-kidney-vascular system diseases and metabolic syndromes (Mets)^1^. A meta-analysis in 2015 reported that the pooled prevalence of HUA is 13.3% in China^2^. A national survey in the US found that the prevalence of HUA is substantial, with 20.2% in males and 20.0% in females aged 20 years and older^3^. HUA has become the second most common metabolic disorder after diabetes mellitus^1^.

The known risk factors for HUA include genetic and environmental factors as well as interactions between them^1^. Recent genome-wide association studies (GWAS) have identified significant associations between single nucleotide polymorphisms (SNPs) in *ABCG2* and *SLC2A9* in HUA cases^4^. Studies have shown that a mutation at the rs2231142 locus of *ABCG2* can cause a 53% reduction in *ABCG2*-mediated serum uric acid (SUA) transport^5^. It is reported that the variation at the rs11722228 locus of *SLC2A9* can also affect the transport and reabsorption of SUA, and the rs11722228 locus of *SLC29* can explain 1.03% of the variation in SUA levels in the Chinese population^6^.

Along with inherited genetic variants, specific dietary components also play a significant role in the development of HUA^7^. Previous studies have reported that red meat, seafood, and alcohol consumption are associated with the risk of HUA^8,9^. However, most of the existing research focuses on the effects of a single food or nutrient rather than the interaction between multiple food items, which may be more relevant to the study of disease association^10,11^. The dietary pattern method uses complex combinations of foods and nutrients to investigate the association between multiple food items and disease^12^. A cross-sectional study found that the ‘animal and fried foods’ pattern was associated with a higher prevalence of HUA^13^, while another study demonstrated that there was no significant association between dietary patterns and HUA in the Chinese population^11^. Hence, the association between dietary patterns and HUA remains unclear.

Potential gene-environment interactions are key features in the development of complex diseases, including HUA and gout^14^. Evidence has suggested that the intake of sugar-sweetened beverages and *SLC2A9* variants interact in the pathogenesis of gout^15^. However, there are few studies on diet and gene interaction in HUA^7,14–16^. To our best knowledge, no study has examined the relationship of dietary patterns and gene interaction with the risk of HUA among the Chinese population.

The Liangshan Yi Autonomous Prefecture is located in southwestern Sichuan province, where the indigenous Yi ethnic group maintains its unique dietary culture^17^. Our previous study found that the prevalence of HUA among adult Yi people was 22%, which was significantly higher than that in the other areas of China^18^. Therefore, we aimed to identify and examine the association between dietary patterns and risk of HUA, as well as to explore whether there is an interaction effect of dietary patterns and gene on the risk of HUA among the adult of Yi population in China.

## Methods

### Study design and population

A cross-sectional study was conducted among the Yi people in the Liangshan District, Sichuan Province, China, from July 2014 to February 2016. A randomized sample of 3188 participants aged 18 and older was obtained using a multistage stratified cluster sampling method. Details of the study design were described in our previous study^19^. In the analysis, we excluded participants who had unretained blood samples and those with incomplete dietary questionnaires. We also excluded participants with cardiovascular diseases or those with medication use that may affect serum uric acid concentration. Therefore, the final study sample size was 2646. The study protocol was approved by the Ethics of Research Committee of Southwest Medical University. All survey methods were performed in accordance with relevant guidelines and regulations. All participants provided informed consent.

### Questionnaire survey

The on-site survey was conducted by well-trained investigators through face-to-face interviews. Participants were asked to complete a questionnaire that included demographic and lifestyle information. Dietary intake was assessed using a semi-quantitative 52-item food frequency questionnaire (FFQ) that inquired on the types of food intake, frequency (daily, weekly, monthly, yearly, or never), and the amount of food consumed over the past year (serving size per gram). The FFQ was designed based on culture-specific dishes, tested using a local sample to check and validate its applicability for the Yi culture. Alcohol consumption was calculated based on the frequency of drinking and consumption of different alcoholic beverages.

### Physical examination

Height and weight were measured by trained investigators to the nearest 0.1 cm and 0.01 kg. Body mass index (BMI) was calculated by weight/height^2^ (kg/m^2^). Blood pressure (BP) was measured twice from the upper left arm after 5 min rest in a seated position. Waist circumference (WC) was measured with a non-retractable tape measure to the nearest 0.1 cm at the umbilical level, with the participants standing and breathing normally. Hip circumference was measured to the nearest 0.1 cm around the symphysis pubis and the posterior gluteus maximus.

### Assessment of hyperuricemia and other biomarkers

HUA was defined as serum uric acid (SUA) concentrations > 420 μmol/L (7.0 mg/dL) for males and > 360 μmol/L (6.0 mg/dL) for females^1^. Overnight fasting blood samples were collected from each participant. SUA, triglycerides (TG), total cholesterol (TC), fasting plasma glucose (GLU), high-density lipoprotein cholesterol (HDL-C), and low-density lipoprotein cholesterol (LDL-C) were measured using an automatic biochemical analyzer (Mindary, BS-820, Shenzhen, China).

### Genomic DNA extraction and genotyping

Genomic DNA was extracted from peripheral blood leukocytes, and a Nano Drop 2000 ultraviolet spectrophotometer was used to measure its quantity and quality. Genotyping was performed by Kompetitive Allele Specific PCR (KASP). The detection success rates of the *ABCG2* rs2231142 and *SLC2A9* rs11722228 loci were 99% and 97.2%, respectively. The SNPs were selected based on the following criteria: (1) SNP with *P* < 1.0 × 10^−5^ for all GWAS samples; (2) when multiple SNPs had a strong LD (r^2^ ≥ 0.8), SNPs previously reported in the literature were used prior to selection; (3) clear genotyping clusters, and (4) Minor Allele Frequency (MAF) ≥ 0.05. Based on quality control criteria, SNPs were excluded when the *P* < 0.001 for the Hardy–Weinberg equilibrium (HWE) test, MAF < 0.01, or genotype call rate < 95%.

### Classification of dietary patterns

According to the Food Composition Table, the similarity of nutrient profiles, and purine contents, we collapsed 52 food items into 17 food groups. The Kaiser–Meyer–Olkin (KMO) measure of sampling adequacy and Bartlett’s sphere test were used to evaluate the adequacy of correlation matrices with the data. Principal component analysis (PCA) and factor analysis (FA) were applied to classify dietary patterns. Varimax rotation was applied to maximize high- and low-value factor loadings and minimize mid-value factor loadings. After evaluation of eigenvalues > 1, the cumulative contribution rate, and the scree plot, three factors were determined. Food items with a factor loading > 0.30 (absolute value) were regarded as the main components of each pattern. The higher the factor loading of a food group, the greater the contribution of that group to the pattern. The labeling of dietary patterns was based on the interpretation of foods with high factor loadings for each pattern^20^. Dietary pattern scores were calculated by summing the product of the standardized intake for each group multiplied by the regression coefficients before they were categorized into quartiles (Qs).

### Statistical analyses

The factor scores of each dietary pattern were divided into quartiles, with Q4 and Q1 the highest and lowest categories for the intake of each dietary pattern, respectively. The association between the quartile categories of dietary pattern scores and HUA was examined using logistic regression analysis. Data for continuous variables are presented as mean ± standard deviation. Categorical variables were generally reported as the sum (percentage) and tested using Chi-square test. The Kaiser–Meyer–Olkin (KMO) measure of sampling adequacy (0.727) and Bartlett’s test of sphericity (*P* < 0.001) showed that the factor model as a whole was significant. The additive interaction was measured using the Delta method described by Rothman et al.^21^, while the parameter estimates and covariance matrix of the logistic regression model were substituted in the Excel program developed by Andersson et al.^22^ to calculate the additive interaction index. The indicators used for evaluating the additive interaction included the excess relative risk (RERI), attributable proportion (AP), and synergy index (S). If the 95% CI of RERI and AP were > 0, and the 95% CI of S > 1, there was a synergistic interaction between the dietary pattern and the gene in HUA. Data were analyzed using SPSS software (SPSS Inc. Version 24). All P values were two-tailed, and the difference was considered significant when *P* < 0.05.

## Results

### Characteristics of participants

**Figure 1:**
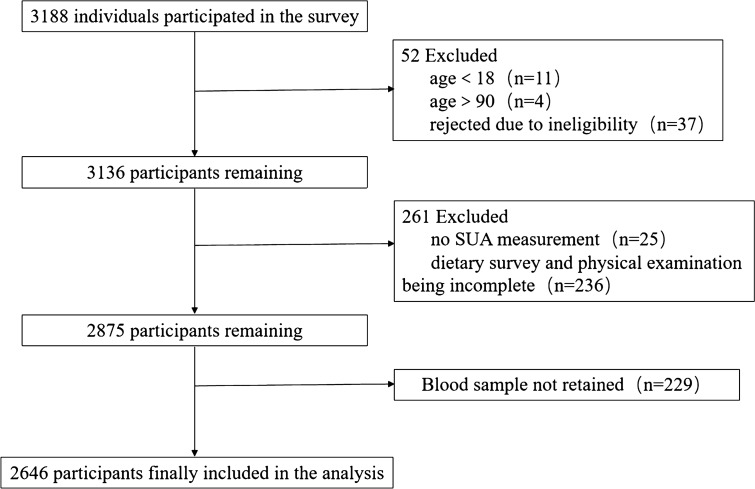
Participant flowchart.

Figure 1 shows the participant flowchart. Of the total analysis sample of 2646 participants (1445 males and 1201 females), the prevalence of HUA was 26.8% in both genders, 34.9% in males, and 17.2% in females. There were significant differences in age, gender, ethnicity, education, and occupation between subjects with and those without HUA. Compared with those without HUA, subjects with HUA had significantly higher means of SUA, TC, and TG, as well as higher WC, WHR, BMI, and BP (Table 1).

**Table 1:** Characteristics of the participants.

Variables	Participants with HUA	Participants without HUA	*P*- value
	n = 710	n = 1936	
Age (years)	42.70 ± 15.06	44.62 ± 14.09	0.003
Gender (%)			** < 0.001**
Male	504 (71.0)	941 (48.6)	
Female	206 (29.0)	995 (51.4)	
Ethnic groups (%)			** < 0.001**
Han	351 (49.4)	687 (35.5)	
Yi	359 (50.6)	1249 (64.5)	
Education (%)			** < 0.001**
≤ Primary school	276 (38.9)	1142 (59.0)	
≥ Junior high school	434 (61.1)	794 (41.0)	
Occupation (%)			** < 0.001**
Farmer	251 (35.6)	1046 (54.3)	
Non-farmer	454 (64.4)	882 (45.7)	
WC (cm)	84.53 ± 10.15	78.49 ± 9.21	** < 0.001**
WHR	0.89 ± 0.06	0.86 ± 0.06	** < 0.001**
BMI (Kg/m^2^)	24.21 ± 3.60	22.43 ± 3.11	** < 0.001**
SBP (mmHg)	131.94 ± 19.49	126.45 ± 18.57	** < 0.001**
DBP (mmHg)	80.91 ± 15.82	77.13 ± 13.97	** < 0.001**
SUA (μmol/L)	466.40 ± 72.71	306.27 ± 60.61	** < 0.001**
LDL (mmol/L)	3.08 ± 0.84	2.92 ± 0.80	** < 0.001**
HDL (mmol/L)	1.25 ± 0.32	1.35 ± 0.33	** < 0.001**
GLU (mmol/L)	5.51 ± 1.39	5.51 ± 1.54	**0.985**
TC (mmol/L)	5.10 ± 1.02	4.93 ± 1.01	** < 0.001**
TG (mmol/L)	2.01 ± 1.45	1.40 ± 1.15	** < 0.001**

*WHR* waist–hip ratio, *SBP* systolic blood pressure, *DBP* diastolic blood pressure, *GLU* serum glucose, *TC* serum total cholesterol, *TG* serum triglycerides. *LDL-cholesterol* low-density lipoprotein cholesterol, *HDL-cholesterol* high-density lipoprotein cholesterol.

Significant values are in bold.

### Dietary patterns

Three major dietary patterns were identified by factor analysis among the participants (Table 2). Factor 1 was defined as the meat-based dietary pattern and characterized by a high intake of animal organ meats, seafood, fresh meat, and eggs; factor 2 was characterized as a plant-based dietary pattern and included the intake of mushroom algae, beans, and their products, nuts, and fruits; and factor 3, was the local special dietary pattern was characterized by the intake of marinated smoked meat and grease. These three patterns accounted for 33·87% of the total variance, with the ‘meat-based,’ ‘plant-based,’ and ‘local special’ dietary patterns constituting 12.64%, 12.54%, and 8.69% of the total variance, respectively.

**Table 2:** Factor loading matrix for classifying dietary patterns.

Food groups	Meat-based	Plant-based	Local special
Viscera	**0.577**	–	–
Snacks and pastries	**0.539**	–	–
Fish, shrimp, crab and shellfish	**0.537**	–	–
Fresh meat	**0.521**	–	–
Eggs	**0.505**	–	–
Cereals	**0.457**	–	–
Grain	**0.451**	–	–
drinks	**0.357**	–	–
Mushrooms and algae	–	**0.716**	–
Beans and their products	–	**0.640**	–
Nuts	–	**0.520**	–
Fruits	–	**0.503**	–
Vegetables	–	**0.487**	–
Milk and dairy products	–	**0.396**	–
Marinated and smoked meat	–	–	**0.727**
Grease	–	–	**0.588**
Alcoholic beverages	–	–	–
Contribution rate (%)	12.640	12.542	8.686
The cumulative contribution rate (%)	12.640	25.182	33.869

Factor loading > 0.30 are listed.

Significant values are in bold.

### Association between dietary patterns and HUA

Table 3 shows that subjects with the meat-based dietary pattern had a significantly high prevalence of HUA. Compared to those in the lowest quartile of the dietary pattern scores, the crude OR of HUA for those in the highest quartile was 1.878 (95% CI = 1.461–2.415, *P* < 0.001). This association remained significant after adjustment for age, BMI, gender, and ethnicity (OR = 1.389, 95% CI: 1.039–1.858, *P* = 0.027). There were no significant associations of the other two dietary patterns with the risk of HUA after these adjustments.

**Table 3:** Association between dietary patterns and HUA.

Dietary pattern	Quartile	Crude model		Adjusted model	
		OR (95% CI)	*P* value	OR (95% CI)	*P* value
Meat-based	Q_1_	1		1	
	Q_2_	1.446 (1.118–1.870)	0.005	1.291 (0.980–1.701)	0.069
	Q_3_	1.644 (1.275–2.119)	** < 0.001**	1.277 (0.959–1.702)	0.094
	Q_4_	1.878 (1.461–2.415)	** < 0.001**	1.389 (1.039–1.858)	0.027
Plant-based	Q_1_	1		1	
	Q_2_	1.119 (0.875–1.432)	0.371	1.184 (0.903–1.553)	0.220
	Q_3_	1.076 (0.841–1.379)	0.559	0.992 (0.757–1.300)	0.953
	Q_4_	1.268 (0.994–1.617)	0.056	1.139 (0.864–1.502)	0.355
Local special	Q_1_	1		1	
	Q_2_	0.932 (0.735–1.181)	0.558	1.066 (0.829–1.370)	0.619
	Q_3_	0.871 (0.686–1.105)	0.255	1.132 (0.872–1.468)	0.351
	Q_4_	0.635 (0.495–0.815)	** < 0.001**	0.874 (0.663–1.151)	0.338

Crude model: without covariates are adjusted. Adjusted model: adjustment for age, BMI, gender, and ethnicity.

Significant values are in bold.

### Hardy–Weinberg equilibrium test and genotype frequency distribution

The allele frequencies for both SNPs in the *ABCG2* case group ($\chi^{2}$ = 0156, *P* = 0.925), control group ($\chi^{2}$ = 0.303, *P* = 0.859), *SLC2A9* case group ($\chi^{2}$ = 0.156, *P* = 0.925), and control group ($\chi^{2}$ = 0.683, *P* = 0.711) were not significant in Hardy–Weinberg equilibrium (*P* > 0.05). The results reveal that the participants analyzed in the study had an adequate group representation. Table 4 displays the genotype frequency distribution of the *ABCG2* and *SLC2A9* genes. For both loci, the T allele in the HUA group was higher than the non-HUA group.

**Table 4:** Frequency distribution of *ABCG2* rs2231142 and *SLC2A9* rs11722228 loci.

SNP sites	Genotype/Allele	HUA group	Non-HUA group	*P* value
rs2231142	G:G	374 (52.7)	1349 (69.7)	** < 0.001**
	G:T	278 (392)	528 (27.3)	
	T:T	58 (8.2)	59 (3.0)	
	G	1026 (72.3)	3226 (83.3)	** < 0.001**
	T	394 (27.7)	646 (16.7)	
rs11722228	C:C	301 (42.4)	904 (46.7)	0.074
	C:T	328 (46.2)	854 (44.1)	
	T:T	81 (11.4)	178 (9.2)	
	C	930 (65.5)	2662 (68.8)	0.025
	T	490 (34.5)	1210 (31.2)	

Significant values are in bold.

### Different genotypes and HUA

**Figure 2:**
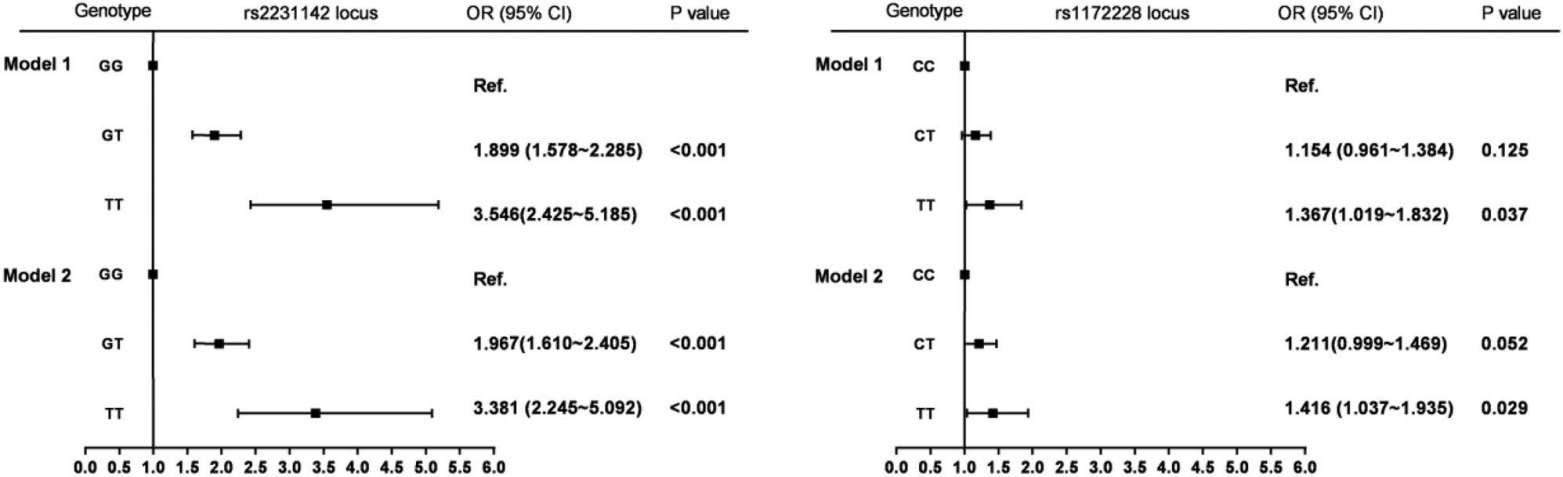
The relationship of *ABCG2* rs2231142 and *SLC2A9* rs11722228 loci with risk of HUA. Model 1 Crude mode. Model 2: adjusted for age, gender, BMI, ethnicity, occupation and education level.

Figure 2 shows that compared to the GG wild-type genotype at the rs2231142 locus in *ABCG2*, the risk of HUA increased with the number of T alleles. Compared to the GG wild-type genotype, the risk of HUA in subjects with GT and TT mutant genotypes was 1.899 times higher (95% CI: 1.578–2.285) and 3.546 times higher (95% CI: 2.425–5.185). After adjustment for age, gender, BMI, ethnicity, occupation, and educational level, the association between the rs2231142 locus and HUA was still significant (*P* < 0.001).

A similar relationship was observed in the rs11722228 locus in *SLC2A9*. Compared with the CC wild-type genotype, the CT and TT mutant genotypes increased the risk of HUA by 21.1% (OR = 1.211, 95% CI: 0.999–1.469) and 41.6% (OR = 1.416, 95% CI: 1.037–1.935) after adjustment for the confounders.

### Interaction analysis between dietary patterns and genetics

There was no evidence that the three dietary patterns had multiplication interaction effects on HUA risk in combination with the *ABCG2* rs2231142 and *SLC2A9* rs11722228 loci. Therefore, we further examined whether there were additive interaction effects of dietary patterns and genes on the risk of HUA.

**Figure 3:**
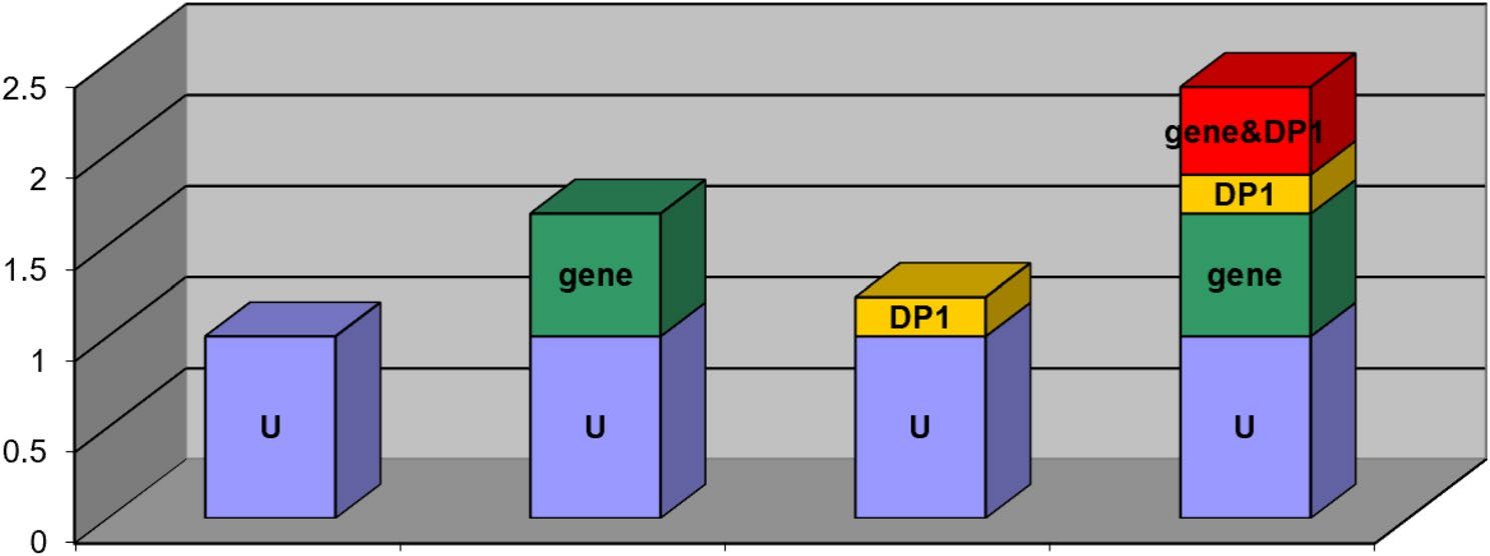
Additive interaction between the ‘Meat-based’ pattern and *ABCG2* rs2231142 locus. U: Baseline; gene: *ABCG2* rs2231142locus; DP1: Dietary pattern 1 (Meat-based pattern); gene&DP1: Additive interaction between the ‘Meat-based’ pattern and *ABCG2* rs2231142 locus.

In the analysis, three dietary pattern scores were dichotomized as two quantiles, with the low quantile as the reference and the rs2231142 locus referenced with the GG wild-type genotype. There was an additive interaction between meat-based dietary pattern and the rs2231142 locus on the risk of HUA; the RERI, AP, and S were 0.482 (95% CI: 0.012–0.976), 0.203 (95% CI: 0.033–0.374), and 1.544 (95% CI: 1.012–2.355), respectively (Table 5, Fig. 3). The other two dietary patterns had no additive interactions with the rs2231142 locus, with 95% CIs of RERI and AP including 0, and the interaction coefficient S including 1. Using the CC wild-type genotype as a reference, the results showed no additive interaction between the rs11722228 locus and the three dietary patterns.

**Table 5:** Additive interaction of dietary patterns with *ABCG2* rs2231142 and *SLC2A9* rs11722228 loci.

SNP site	Dietary patterns	RERI	AP	S
rs2231142	Meat-based	0.482 (0.012–0.976)	0.203 (0.033–0.374)	1.544 (1.012–2.355)
	Plant-based	0.049 (− 0.421–0.519)	0.022 (− 0.183–0.227)	1.041 (0.713–1.520)
	Local special	− 0.140 (− 0.496–0.215)	− 0.099 (− 0.353–0.156)	0.750 (0.367–1.534)
rs11722228	Meat-based	0.024 (− 0.551–0.600)	0.009 (− 0.208–0.226)	1.015 (0.713–1.446)
	Plant-based	− 0.033 (− 0.367–0.302)	− 0.022 (− 0.244–0.201)	0.941 (0.501–1.764)
	Local special	− 0.052 (− 0.298–0.194)	− 0.055 (− 0.315–0.205)	14.979 (0.000–33.426)

**Figure 4:**
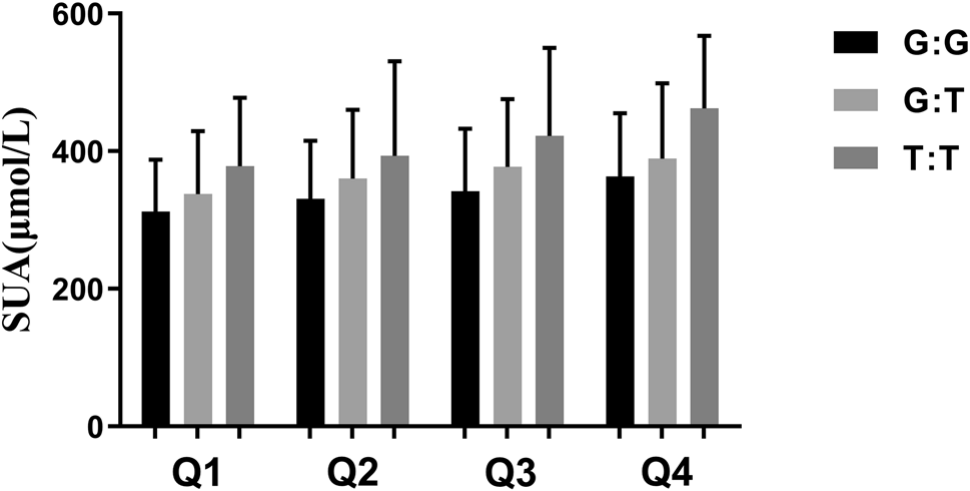
The SUA level of *ABCG2* rs2231142 locus in ‘Meat-based’ pattern quartile.

When the average SUA levels of each genotype at the *ABCG2* rs2231142 locus were compared under the "animal diet pattern" quartile, that SUA levels increased with the number of T risk alleles (GG → GT → TT). In addition, the SUA level increased as the "meat-based dietary pattern" factor score increased in the same genotype (Fig. 4).

## Discussion

In this study, three dietary patterns were identified among the Yi people who lived in the Liangshan District of Sichuan Province, China. These three dietary patterns were meat-based, plant-based, and specific cuisine. The main finding of this study is that the meat-based food pattern, which is characterized by a high intake of animal organ meats, seafood, fresh meat, and eggs, was positively associated with an elevated risk of HUA, whereas no significant association was observed between the other two dietary patterns and HUA risk.

To date, several studies have explored the association between diet and HUA. A cross-sectional study demonstrated that animal products and fried food patterns that are rich in pork, eggs, animal giblets, poultry, and fried wheat, was positively associated with a higher prevalence of HUA^13^. He et al.^23^ found that a meat food pattern characterized by the intake of poultry, beef, processed and cooked meat, eggs, and fats, was associated with an elevated risk of HUA. In contrast, a study in Taiwan showed that after adjusting for age, gender, and body mass index, the significant level of the relationship between the dietary pattern and HUA were attenuated^11^. Another study suggested that compared with individuals in the lowest quintile, those in the highest quintile of meat and seafood intake experienced an increased risk of gout^8^. Our results also show that the proportion of alcohol consumption in those with HUA was much higher than that in those without HUA^24^. Alcohol consumption may increase the risk of HUA. The higher rate of alcohol consumption was largely related to the local lifestyles where people like to have alcohol consumption^17^.

Our study observed a positive and significant association between the meat-based dietary pattern and the prevalence of HUA. This finding is consistent with previous reports^13,23^. The mechanism that has been proposed to explain this association is that animal meat-based foods contain high purines. Excessive intake of purines would lead to an increase in SUA levels, subsequently increasing the risk of HUA^8^. Our previous study found that people in the Liangshan area are more likely to drink broth, and the meat consumed is mainly fresh pork fat^17^, which contains more purines and saturated fatty acids. The consumption of animal foods, especially the calories and fat in red meat, leads to centripetal obesity that is an established factor for an increase in plasma insulin levels. Studies have shown that plasma insulin concentrations are significantly associated with HUA^13,25^. A previous study observed that a dietary pattern with increased soybean product and fruit intakes was negatively associated with the prevalence of HUA^13^. Fruits and vegetables can provide rich dietary fiber and their antioxidant components, which have protective effects against HUA^10^. However, in the study, we did not observe a significant association of plant-based and local dietary patterns with a risk reduction of HUA. We are unable to explain this finding using the current dataset. Further research is warranted.

The heritability of SUA concentration is approximately 40–70%^26^. Previous GWAS studies suggested an association between HUA susceptibility relative to dysfunctional *ABCG2* variants, with the rs2231142 locus being the most common variant^27^. In our study, we found that individuals with the *ABCG2* rs2231142 T allele had a higher frequency of HUA and SUA than those with the rs2231142 GG allele. This finding was consistent with results reported in the United States^28^ and in Mexico^29^. *ABCG2* is a high-capacity urate transporter that plays a key role in renal urate overload^27^. The risk of HUA that is attributed to *ABCG2* variants is 29.2%, which is much higher than other typical environmental risks^30^. Our findings suggest that the rs2231142 locus variants in *ABCG2 might be* extremely important in the pathogenesis of HUA. This result supports the important strategy of *ABCG2* genotyping applied in the screening of individuals with a high risk of HUA^27^.

The *SLC2A9* gene encodes two GLUT9 isoforms of class II facilitative glucose transport family^7^. A previous study reported that the rs11722228 locus of *SLC29* can explain 1.03% of the variation in SUA levels in the Chinese population^6,31^. Doring et al. reported that the most significant SNPs associated with SUA were within the *SLC2A9* gene^32^. Our study also found that the SUA level in individuals with the rs11722228 locus showed increased instances of the T allele and was higher in the HUA population than in the non-HUA population.

Longitudinal studies have found that some individuals are more sensitive to unhealthy diets, reflecting the complex interaction between genetic factors and diet^14^. In this study, we found an additive interaction between a meat-based dietary pattern and the rs2231142 locus of *ABCG2*. Under the same dietary pattern factor score, the increase of the T allele at the rs2231142 locus was associated with increased SUA levels. Moreover, individuals with this genotype also exhibited SUA levels that increased with the dietary pattern factor score. Similar to our results, Beydoun et al.^7^ reported that 12 SNP sites (including *ABCG2* and *SLC2A9*) are closely related to SUA and found that there was a synergistic interaction between the genetic risk score (GRS) and red meat intake that increased the risk of increased SUA among women. In addition, a population-based study in Malaysia^33^ showed that SUA concentration is affected by the interaction of a ‘fruit diet pattern’ and the *VEGFR-2* rs2071559 locus, suggesting that fruit consumption may play a role in enhancing or suppressing the effects of *VEGFR-2* polymorphisms. Studies have shown that people of Han-Chinese could be inherently predisposed to higher risk for HUA and gout compared with other populations, which may partly explain the finding of no association between a plant-based diet and the ABCG2^34^.

To the best of our knowledge, there are four main studies that examined the interaction effect of diet and gene expression on HUA^7,14–16^. These results support the hypothesis that different dietary intakes can enhance or weaken the impact of genes on blood UA level and HUA. However, only one study included the rs2231142 and rs11722228 loci^7^, and none of these studies were conducted among a Chinese population. Our study used a large sample size and careful study design to add new evidence of diet and genetic interactions on HUA prevalence in the Chinese people to the field. The findings of this study will provide new and necessary insight into HUA and assist in developing intervention strategies.

Most studies on the interaction between diet and genetics in China have focused on diseases such as Mets^35^ and obesity^36^. Our study is among the few to explore the influence of dietary patterns and gene interactions on HUA. We also evaluated dietary patterns using FFQ, which consists of purine-containing foods and beverages commonly consumed by the Chinese population. This is important because the dietary structure is a key factor for assessing the prevalence of HUA within populations^37^. In addition, information from the questionnaires was collected by trained investigators, and thus our results are reliable.

This study had several limitations. First, it is a cross-sectional study, and the SUA level was only a one-time measurement. Second, the information about food consumption may have recall bias. Although we adjusted for potential confounding variables, we were unable to control for the effect of unmeasured confounding factors. Third, we only focused on two SNPs closely related to HUA. There could be other SNPs in different genes that may also contribute to diet-gene interactions, limiting analyses to these two genes could have biased the results. Therefore, more SNPs will be considered in further research.

In conclusion, a higher adherence to a meat-based dietary pattern was associated with a higher prevalence of HUA in the Yi ethnic group of the Chinese population. There was a significant additive interaction between the meat-based dietary pattern and the *ABCG2* rs2231142 locus on the risk of HUA. The findings of the study provide new insights into intervention strategies and health policy for controlling the risk of HUA among the Chinese population. Further prospective studies are needed to confirm these findings.

## Data Availability

Data described in the manuscript, codebook, and analytic codes cannot be made available due to the University Institution Review Board requirement.

## References

[1] Multidisciplinary Expert Task Force on Hyperuricemia and Related Diseases (2017). Chinese multidisciplinary expert consensus on the diagnosis and treatment of hyperuricemia and related diseases. Chin. Med. J..

[2] Liu R, Han C, Wu D, Xia X, Gu J, Guan H (2015). Prevalence of hyperuricemia and gout in mainland China from 2000 to 2014: A systematic review and meta-analysis. Biomed. Res. Int..

[3] Chen-Xu M, Yokose C, Rai SK, Pillinger MH, Choi HK (2019). Contemporary prevalence of gout and hyperuricemia in the United States and decadal trends: The national health and nutrition examination survey, 2007–2016. Arthritis Rheumatol.

[4] George RL, Keenan RT (2013). Genetics of hyperuricemia and gout: Implications for the present and future. Curr Rheumatol Rep.

[5] Woodward OM, Köttgen A, Coresh J, Boerwinkle E, Guggino WB, Köttgen M (2009). Identification of a urate transporter, ABCG2, with a common functional polymorphism causing gout. Proc. Natl. Acad. Sci. USA.

[6] Yang B, Mo Z, Wu C, Yang H, Yang X, He Y (2014). A genome-wide association study identifies common variants influencing serum uric acid concentrations in a Chinese population. BMC Med. Genomics.

[7] Beydoun MA, Canas JA, Fanelli-Kuczmarski MT, Tajuddin SM, Evans MK, Zonderman AB (2017). Genetic risk scores, sex and dietary factors interact to alter serum uric acid trajectory among African-American urban adults. Br. J. Nutr..

[8] Choi HK, Atkinson K, Karlson EW, Willett W, Curhan G (2004). Purine-rich foods, dairy and protein intake, and the risk of gout in men. N. Engl. J. Med..

[9] Choi HK, Atkinson K, Karlson EW, Willett W, Curhan G (2004). Alcohol intake and risk of incident gout in men: A prospective study. Lancet.

[10] Zykova SN, Storhaug HM, Toft I, Chadban SJ, Jenssen TG, White SL (2015). Cross-sectional analysis of nutrition and serum uric acid in two caucasian cohorts: The AusDiab Study and the Tromsø study. Nutr. J..

[11] Tsai YT, Liu JP, Tu YK, Lee MS, Chen PR, Hsu HC (2012). Relationship between dietary patterns and serum uric acid concentrations among ethnic Chinese adults in Taiwan. Asia Pac. J. Clin. Nutr..

[12] Hu FB (2002). Dietary pattern analysis: A new direction in nutritional epidemiology. Curr. Opin. Lipidol..

[13] Zhang M, Chang H, Gao Y, Wang X, Xu W, Liu D (2012). Major dietary patterns and risk of asymptomatic hyperuricemia in Chinese adults. J. Nutr. Sci. Vitaminol. (Tokyo).

[14] Rasheed H, Stamp LK, Dalbeth N, Merriman TR (2017). Interaction of the GCKR and A1CF loci with alcohol consumption to influence the risk of gout. Arthritis Res. Ther..

[15] Batt C, Phipps-Green AJ, Black MA, Cadzow M, Merriman ME, Topless R (2014). Sugar-sweetened beverage consumption: a risk factor for prevalent gout with SLC2A9 genotype-specific effects on serum urate and risk of gout. Ann. Rheum. Dis..

[16] Roseline YW, Shidoji Y, Hon WM, Masaki M (2012). Association and interaction effect between VEGF receptor-2 (VEGFR-2) gene polymorphisms and dietary pattern on blood uric acid in Malays and Indians. Malays. J. Nutr..

[17] Liu X, Huang S, Xu W, Zhou A, Li H, Zhang R (2018). Association of dietary patterns and hyperuricemia: A cross-sectional study of the Yi ethnic group in China. Food Nutr. Res..

[18] Zhou AJ, Pan Q, Li AL, Qie YL, Wang QX, Gong Y (2015). Predictive value of obesity and metabolism indexes for hyperuricemia among rural adult Yi residents in Liangshan region. Chin. J. Pub. Health.

[19] Huang S, Liu X, Li H, Xu W, Jia H (2017). Sex difference in the association of serum uric acid with metabolic syndrome and its components: A cross-sectional study in a Chinese Yi population. Postgrad. Med..

[20] Newby PK, Tucker KL (2004). Empirically derived eating patterns using factor or cluster analysis: A review. Nutr. Rev..

[21] Rothman KJ (2002). Epidemiology: An Introduction.

[22] Andersson T, Alfredsson L, Källberg H, Zdravkovic S, Ahlbom A (2005). Calculating measures of biological interaction. Eur. J. Epidemiol..

[23] He F, Wang LL, Yu XL (2017). Dietary patterns associated hyperuricemia among Chinese aged 45–59 years: An observational study. Medicine.

[24] Ya, L., Q, W., Aijing, Z., et al. A comparative study on health-related behaviors between Yi and Han in Liangshan, Sichuan. *Mod. Prev. Med.* **19**, 3531 3533+3540. CNKI:SUN:XDYF.0.2015-19-025.

[25] Shu L, Zheng PF, Zhang XY, Si CJ, Yu XL, Gao W (2015). Association between dietary patterns and the indicators of obesity among Chinese: A cross-sectional study. Nutrients.

[26] Köttgen A, Albrecht E, Teumer A, Vitart V, Krumsiek J, Hundertmark C (2013). Genome-wide association analyses identify 18 new loci associated with serum urate concentrations. Nat. Genet..

[27] Stiburkova B, Pavelcova K, Pavlikova M, Ješina P, Pavelka K (2019). The impact of dysfunctional variants of ABCG2 on hyperuricemia and gout in pediatric-onset patients. Arthritis Res. Ther..

[28] Dehghan A, Köttgen A, Yang Q, Hwang SJ, Kao WL, Rivadeneira F (2008). Association of three genetic loci with uric acid concentration and risk of gout: A genome-wide association study. Lancet.

[29] Rivera-Paredez B, Macías-Kauffer L, Fernandez-Lopez JC, Villalobos-Comparán M, Martinez-Aguilar MM, de la Cruz-Montoya A (2019). Influence of genetic and non-genetic risk factors for serum uric acid levels and hyperuricemia in mexicans. Nutrients.

[30] Nakayama A, Matsuo H, Nakaoka H, Nakamura T, Nakashima H, Takada Y (2014). Common dysfunctional variants of ABCG2 have a stronger impact on hyperuricemia progression than typical environmental risk factors. Sci. Rep..

[31] Kamatani Y, Matsuda K, Okada Y (2010). Genome-wide association study of hematological and biochemical traits in a Japanese population. Nat. Genet..

[32] Döring A, Gieger C, Mehta D, Gohlke H, Prokisch H, Coassin S (2008). SLC2A9 influences uric acid concentrations with pronounced sex-specific effects. Nat. Genet..

[33] Roseline YW, Shidoji Y, Hon WM (2012). Association and interaction effect between VEGF receptor-2 (VEGFR-2) gene polymorphisms and dietary pattern on blood uric acid in Malays and Indians. Malays. J. Nutr..

[34] Butler F, Alghubayshi A, Roman Y (2021). The Epidemiology and genetics of hyperuricemia and gout across major racial groups: A literature review and population genetics secondary database analysis. J. Pers. Med..

[35] Ching YK, Chin YS, Appukutty M, Ramachandran V, Yu CY, Ang GY (2019). Interaction of dietary linoleic acid and α-linolenic acids with rs174547 in FADS1 gene on metabolic syndrome components among vegetarians. Nutrients.

[36] Wang T, Xu M, Bi Y, Ning G (2018). Interplay between diet and genetic susceptibility in obesity and related traits. Front. Med..

[37] Zhang X, Meng Q, Feng J, Liao H, Shi R, Shi D (2018). The prevalence of hyperuricemia and its correlates in Ganzi Tibetan Autonomous Prefecture, Sichuan Province China. Lipids Health Dis..

